# Attitudes and training related to substance use in pediatric emergency departments

**DOI:** 10.1186/s13722-022-00339-w

**Published:** 2022-10-23

**Authors:** Ariel M. Hoch, Samantha F. Schoenberger, Tehnaz P. Boyle, Scott E. Hadland, Mam Jarra Gai, Sarah M. Bagley

**Affiliations:** 1 Division of Pediatric Emergency Medicine, Department of Pediatrics, Boston Medical Center, Boston University School of Medicine, One Boston Medical Center Place, Boston, MA 02118 USA; 2 Section of General Internal Medicine, Department of Medicine, Boston Medical Center, Boston University School of Medicine, Boston, MA USA; 3 Grayken Center for Addiction, Boston Medical Center, Boston, MA USA; 4 Division of General Pediatrics, Department of Pediatrics, Boston Medical Center, Boston University School of Medicine, Boston, MA USA; 5 Division of Adolescent Medicine, Department of Pediatrics, MassGeneral Hospital, Harvard Medical School, Boston, MA USA

**Keywords:** Pediatric Emergency Medicine, Substance use disorder, Opioid use disorder

## Abstract

**Background:**

In recent years, pediatric emergency departments (PED) have seen an increase in presentations related to substance use among their adolescent patient population. We aimed to examine pediatric emergency medicine (PEM) physicians’ knowledge, attitudes, and beliefs on caring for adolescents with substance use.

**Methods:**

We conducted a cross-sectional online survey of PEM physicians through the American Academy of Pediatrics Pediatric Emergency Medicine Collaborative Research Committee (PEM-CRC) listserv. The 41-item survey contained the following domains: demographics, current protocols and education for managing adolescent substance use, and attitudes about treatment of substance use. We calculated descriptive statistics for each variable within the domains.

**Results:**

Of 177 respondents (38.2% response rate), 55.4% were female, 45.2% aged ≥ 50 years, 78% worked in a children’s hospital, and 50.8% had > 15 years clinical practice. Overall, 77.8% reported caring for adolescents with a chief complaint related to non-opioid substance use and 26.0% opioid use at least once a month. Most (80.9%) reported feeling comfortable treating major medical complications of substance use, while less than half were comfortable treating withdrawal symptoms. 73% said that they were not interested in prescribing buprenorphine.

**Conclusions:**

Among this national sample of PEM physicians, 3 of 4 physicians managed substance-related visits monthly, but 52% lacked comfort in managing withdrawal symptoms and 73.1% were not interested in prescribing buprenorphine. Almost all PEM physician identified substance use-related education is important but lacked access to faculty expertise or educational content. Expanded access to education and training for PEM physicians related to substance use is needed.

**Supplementary Information:**

The online version contains supplementary material available at 10.1186/s13722-022-00339-w.

## Background

Substance use and substance use disorder (SUD) are major contributors to morbidity and mortality in the United States [1]. Substance use often begins in adolescence and is associated with short term risks like injury, sexually transmitted infections, and motor vehicle accidents, as well as development of a SUD later in life [2–7].

Leveraging the pediatric emergency department (PED) as a critical touchpoint for adolescents who use substances represents an important potential opportunity. Cannabis-associated ED visits have increased annually from 2006 to 2018, with patients 0–14 years accounting for a significant rate increase in recent years [8]. Alcohol misuse-related ED visits are prevalent, with nearly 40% of substance-related ED visits between 2010 and 2013 in patients aged 12 to 20 involving alcohol misuse [9]. Furthermore, ED visits for opioid-related indications have risen substantially in children over the past two decades, and hospitalizations attributed to opioid poisonings among those aged 19 and younger increasing nearly twofold. [10] Between 2016 and 2019, suspected drug overdoses accounted for 85.2 per 10,000 ED visits in adolescents aged 15–24 years old, and 43.2 per 10,000 among children aged 11–14 years. [11]

As the front doors to healthcare in the United States, PEDs offer the medical community a critical opportunity for responding to the public health challenge related to substance use and drug overdose deaths. Despite an increase in presentations to PEDs for substance use [10, 12], there is a lack of education and guidance for pediatric emergency medicine (PEM) physicians to provide care for this population. As a primary site for managing acute substance intoxication and routine encounters with patients who have substance related issue, including intoxication, withdrawal, injury or overdose, the PED is an underutilized setting to screen, treat, and provide referrals to treatment and harm reduction services [13–15].

In recent years, adult EDs have implemented educational materials and protocols for initiation of opioid use disorder treatment and naloxone distribution by emergency medicine physicians [16, 17]. Previous research indicates that ED physicians support harm reduction approaches, such as naloxone [16, 17]. However, prior work has found barriers to implementation such as lack of knowledge, time, and institutional support [18].

Current recommendations for pediatric practitioners from the American Academy of Pediatrics (AAP) focuses on outpatient settings, including screening, brief intervention, and referral for substance use in primary care settings [19], care for families affected by substance use [20], and referral to opioid use disorder (OUD) treatment and medication treatment [21]. These interventions are appropriate for managing substance use longitudinally, however PEM physicians would benefit from similar clinical guidelines and educational tools to support care in the PED which is more likely to focus on management of acute symptoms and stabilization.

PEM physicians may lack the training and comfort to provide effective care for substance use and consider the pediatric emergency department ill-suited for management and treatment [22, 23]. PEM physician’s training is unique among the pediatric subspecialities, in that physician can enroll in a fellowship after completing a residency in either pediatrics or emergency medicine, with the majority being pediatric trained. The overall number of EM-trained PEM physicians is low and continues to decline [24]. An important gap in pediatric training may be the lack of routine education on substance use and substance use disorders in PEM curricula as compared to adult EM education, as well as physician comfort in treating substance use and related harms. In previous studies, pediatric physicians identified limited time, competing medical problems, physician hesitancy to screen when treatment resources are limited, and a lack of training and thus limited knowledge of screening tools as reasons for not implementing clinical guidelines [25, 26]. Training and comfort in caring for and treating substance use among PEM physicians has been understudied, and with prior research primarily focused on alcohol use and less emphasis on other substances, including opioid use disorder [26].

Given the increased rate of substance-related PED presentations and limited guidance for PEM physicians about managing substance use, we sought to: (1) examine PEM physician education on adolescent substance use, (2) describe attitudes and beliefs of PEM physicians about treatment of substance use, and (3) identify proportion of PEDs with protocols to address substance use.

## Methods

### Respondents

We conducted a cross-sectional survey of pediatric emergency medicine physicians from the AAP Section on Emergency Medicine (SOEM) Pediatric Emergency Medicine Collaborative Research Committee (PEM-CRC). The study was approved by the hospital institutional review board at Boston Medical Center. The survey was distributed online through the PEM-CRC listserv to the 463 section members of the SOEM PEM-CRC (Additional file 1).

### Survey development and administration

The survey instrument was developed by the study investigators and reviewed by the Pediatric Emergency Medicine Collaborative Research Committee’s survey committee. Study questions probed PEM physicians on current protocols and education related to adolescent substance use in PEDs across the United States. The survey draft was distributed to team members, which included two PEM-board certified clinician-investigators and two Addiction Medicine-board certified clinician-investigators.

Survey questions were piloted with five PEM physicians from three different US institutions. Their feedback was incorporated to refine the survey for clarity. The final survey consisted of 41 items and took 10 min based on pilot testing.

The structure of the survey included a variety of multiple choice, Likert-type, ranking, and open-ended questions. There were four domains: (1) demographics (sex, age, years in practice, clinical hours, board certification), (2) participant practice setting (patient volume, primary practice site and setting, frequency of patients with chief complaints related to substance use), (3) procedures/protocols (protocols available, consults available, buprenorphine waiver training), (4) attitudes (physician comfort in treating substance use, importance of adolescent substance use education, rating of current education, barriers to education). Physician comfort in caring for patients with substance use was measured on a 4-point Likert-type scale, ranging from “very comfortable” to “not comfortable”. For the analysis, we created composite variables. We classified a physician as having comfort in caring for patients with substance use if they reported “very comfortable” or “comfortable” on the 4-point Likert scale. Physician’s attitude on the importance of education on adolescent substance use was measured on a 4-point Likert-type scale, ranging from “very important” to “not important”. For the analysis, we created composite variables. We classified physicians reporting they felt it is important to receive training on caring for patients with substance use if they answered “very important” or “important” on the 4-point Likert scale.

The electronic survey link was administered to the PEM-CRC listserv members via e-mail, and study data were collected using REDCap, hosted at Boston University [27]. Participants who were unable to receive the email due to inaccurate e-mail addresses or non-practicing physicians with automated email responses were excluded from survey administration. Distribution occurred during a 3-month period from September 1, 2020 to November 30, 2020. Electronic reminders were sent through e-mail via the listserv at 1 and 2 months after initial distribution. Consent for participation was implied via initiation and completion of the survey as stated at distribution, and all responses were anonymous. After completion, the participants were given an option to voluntarily provide their email for entry into $300 raffle for participation.

### Data collection and analysis

Study data were collected and managed using REDCap electronic data capture tools hosted at Boston University [27]. Data analysis for this paper was generated using SAS software and included descriptive statistics to characterize our study population. Copyright, SAS Institute Inc. SAS and all other SAS Institute Inc. product or service names are registered trademarks or trademarks of SAS Institute Inc., Cary, NC, USA. We used a chi-square test of independence (or Fischer’s exact test when < 5) to compare various factors against physician comfort in treating major medical complications and interest in becoming buprenorphine waivered. Missing values for any variable were excluded from analysis. Outcomes were described using weighted percentages and an alpha level of p < 0.05 was used to determine statistical significance.

## Results

### Respondent characteristics

A total of 177 (38.2%) of the PEM physicians completed the survey. The demographic characteristics of respondents are shown in Table 1. Overall, 55.4% (98/177) were female and 45.2% (80/177) were greater than 50 years old. In total, 78.0% (138/177) of the PEM physicians worked in a PED in a children’s hospital, 85.3% (151/177) were university-affiliated, and 85.9% (152/177) worked in an urban setting. When asked about board certification, 84.2% (149/177) were board certified in Pediatrics, 3.4% (6/177) in Emergency Medicine, and 95.5% (169/177) in Pediatric Emergency Medicine. A total of 68.4% (121/177) reported their annual pediatric ED patient volume of > 40,000, 50.8% (90/177) had been in independent clinical practice for > 15 years, and 33.9% (60/177) worked less than 15 clinical hours per week. Overall, 77.8% (137/177) and 26.0% (46/177) reported caring for adolescent patients who presented with a chief complaint related to non-opioid and opioid substance use, respectively, at least once a month. A total of 14.1% (25/177) physicians reported providing naloxone either through a naloxone kit given at discharge or a prescription for naloxone when there was an opioid-related visit.

**Table 1:** Demographics (N = 177)

	N (%)
Sex/gender	
Male	79 (44.6)
Female	98 (55.4)
Age	
30–40	39 (22.0)
41–50	58 (32.8)
> 50	80 (45.2)
Primary hospital site	
University-affiliated	151 (85.3)
Community-based	23 (13.0)
Other	3 (1.7)
Primary practice site	
Pediatric ED in a children’s hospital	138 (78.0)
Pediatric ED in a general hospital/Urgent care	39 (22.0)
Annual ED pediatric patient volume	
0 to < 40,000	52 (29.4)
≥ 40,000	121 (68.4)
Unknown	4 (2.2)
Setting of primary practice site	
Urban	152 (85.9)
Suburban/rural	25 (14.1)
Location by census regions and divisions	
West	39 (22.0)
Midwest	49 (27.7)
South	42 (23.7)
Northeast	44 (24.9)
Other	3 (1.7)
Hours per week in a clinical capacity	
< 15 per week	60 (33.9)
15–30 per week	94 (53.1)
> 30 per week	23 (13.0)
Years in independent clinical practice	
≤ 15	86 (48.6)
> 15	90 (50.8)
Unknown	1 (0.6)
Board certification (select all)	
Pediatrics	149 (84.2)
Emergency medicine	6 (3.4)
Pediatric emergency medicine	169 (95.5)
Other	7 (4.0)
None	1 (0.6)
Care for adolescent patients with a chief complaint related to non-opioid substances at least once a month	137 (77.8)
Care for adolescent patients with a chief complaint related to non-medical opioid use at least once a month	46 (26.0)

### Physician comfort treating substance use

Physician comfort in caring for patients with substance use is outlined in Table 2. Overall, 80.9% (140/173) of respondents reported feeling comfortable treating major medical complications of substance use and 94.8% (164/173) with managing acute treatment of intoxication. However, 48.0% (83/173) and 42.2% (73/173) felt comfortable treating withdrawal and making referrals for substance use disorder, respectively.

**Table 2:** Provider comfort (N = 173)

	Comfortable^a^, n (%)	Not Comfortable^b^, n (%)
Treating medical complications	140 (80.9)	33 (19.1)
Completing additional medical screening that may be indicated if chief complaint is related to substance use	124 (71.7)	49 (28.3)
Acutely treating intoxication	164 (94.8)	9 (5.2)
Acutely treating withdrawal	83 (48.0)	90 (52.0)
Making a treatment referral for substance use disorder	73 (42.2)	100 (57.8)

^a^For analysis, the composite variable “Comfortable” included “Very Comfortable” or “Comfortable” on the 4-point Likert scale

^b^For analysis, the composite variable “Not Comfortable” included “Neither Comfortable or Uncomfortable” or “Not Comfortable” on the 4-point Likert scale

Participants who reported being comfortable treating major medical complications related to substance use were more likely to have their primary site setting located in an urban environment (p = 0.02). No significant difference in physician comfort was found when comparing subgroups by sex, age, region of practice, hospital site, or annual ED volume.

### Attitudes towards training

The majority of PEM physicians felt it was important to receive training on caring for patients with substance use. In total, 91.9% (158/172) and 93.0% (160/172) felt it is important to have education about adolescent use of non-opioid and opioid substances, respectively. When asked more specifically about what type of training was important to receive, physicians answered the following: 100.0% (172/172) for acute treatment of intoxication, 95.4% (164/172) for acute treatment of withdrawal, and 90.7% (156/172) for treatment referral for substance use disorder. When practicing physicians were asked what barriers exist to receiving physician education on opioid substance use at their primary practice site, they cited lack of faculty expertise (49.1%, 84/172), absence of curricular content (40.0%, 69/172), and lack of curricular time (40.0%, 69/172) as the most significant barriers. A total of 70.1% (124/172) respondents were interested in a web-based curriculum on adolescent opioid misuse.

### Protocols in pediatric emergency departments

With regard to the presence of protocols for managing adolescents who present with a chief complaint related to substance use, 74.6% (132/177) reported no protocols exists in their clinical workplace, and 9.6% (17/177) were unsure. Of the 15.8% (28) respondents reporting the presence of protocols, 37.0% (10/28) related to alcohol withdrawal, 70.4% (19/28) opioid withdrawal, and 40.7% (11/28) acute pain management in patients with opioid use disorder. PEM physicians reported various services at their primary practice site to assist with the care of adolescent patients with substance use disorders, with the majority 85.3% (151/177) having a social worker available, while 19.2% (34/177) had an addiction medicine team available.

### Buprenorphine training

**Fig. 1:**
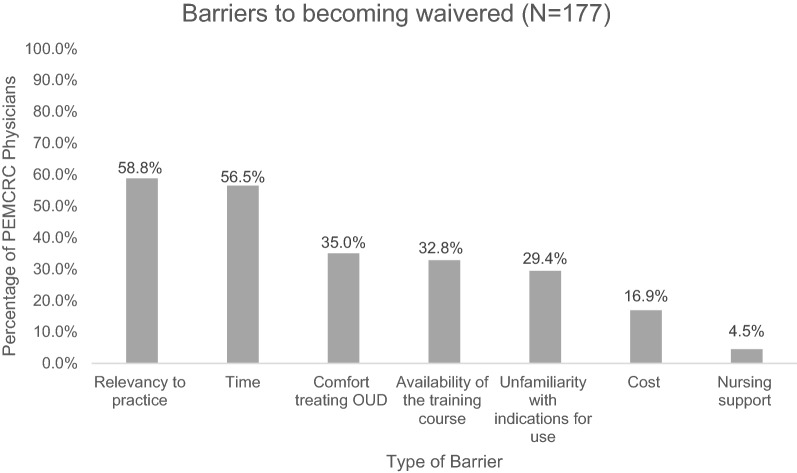
Barriers to becoming buprenorphine waivered

PEM physicians were surveyed on the Drug Addiction Treatment Act of 2000, which requires providers to complete 8 h of qualified training to apply for a waiver (X waiver) to write a prescription to be filled outpatient from the ED setting [28]. A total of 15.8% (28/177) reported access to waiver training for buprenorphine prescribing at their primary site. Only 3.3% (6/177) of the respondents had completed the X-waiver training to prescribe buprenorphine. All six did an online course and have obtained a waiver. Of the 171 who had not completed a waiver course, 73.1% (125/171) were not interested in becoming waivered. Barriers to becoming waivered included time (56.5%, 100/177) and relevancy to practice (58.8%, 104/177) being the most common barriers reported (Fig. 1).

Participants who reported being interested in becoming buprenorphine waivered were more likely to be younger physicians (p = 0.04). No significant difference in physician interest was found when comparing subgroups by sex, region of practice, hospital site, or annual ED volume (Additional file 2).

## Discussion

In this study of PEM physicians conducted through the PEM-CRC, 77.8% and 26.0% reported past month management of adolescents with non-opioid and opioid substance use, respectively. Nearly all survey respondents felt it was important to receive training on care for patients with substance use and expressed interest in access to additional resources to address substance use. However, greater than 50% lacked comfort in managing withdrawal symptoms or making referrals for substance use disorder. A total of 85.9% of PEM physicians surveyed did not provide naloxone when there was an opioid-related visit. Of unwaivered participants, 73.1% were not interested in receiving training to prescribe buprenorphine.

We found that survey respondents were more comfortable treating acute intoxication than treating withdrawal symptoms or making referrals for substance use disorder. This may be because pediatric emergency medicine training and practice focuses on the immediate resuscitation, management, and stabilization of patients, with less emphasis placed on withdrawal management or efforts to help patients initiate treatment. Managing withdrawal symptoms is critical, especially for certain substances in which withdrawal can be life-threatening. Withdrawal treatment aims to minimize symptoms, prevent complications, and create an opportunity to discuss harm reduction and treatment. Future efforts aimed at improving withdrawal management among PEM physicians may lead to substantial improvements in the care of patients with substance use in PEDs.

Our study found that PEM physicians surveyed expressed the importance of education and training on adolescent substance use. The majority reported that they felt training in both opioid and non-opioid substance use is important but did not have adequate resources or access at their institutions. This is important because there are evidence-based, emergency department interventions for adolescent alcohol and cannabis use [29, 30]. It is unknown how well disseminated these interventions are in the community. With lack of faculty expertise, absence of curricular content, and lack of curricular time reported as the most significant barriers to substance use education, future efforts aimed at addressing these knowledge gaps through web-based curricula and focused algorithms and guidelines may help PEM physicians more consistently and appropriately manage substance use disorder in their adolescent patient population.

A potential first step improving knowledge and comfort is strengthening the foundation of training through required substance use education during both Pediatric residency and PEM fellowship. Furthermore, simulation-based training could also be incorporated during fellowship training, with an increased focus on both acute intoxication and withdrawal management. In addition to efforts aimed at trainees, a focus on multidisciplinary collaboration in the development of ED-based protocols and ordersets can help guide in the treatment of intoxication and withdrawal. These protocols can incorporate consultant services such as social work, addiction medicine, adolescent medicine, pain specialists, which will help standardize treatment. Finally, PEDs should engage their community stakeholders in referral mechanisms to improve the continuity of care in their patients with substance use disorder.

Given the rising overdose deaths among youth, there is an urgent need to offer evidence-based interventions including naloxone and treatment with medications for opioid use disorder such as buprenorphine. Prior data suggests that among adolescents aged 14 to 18 years old, fentanyl-involved fatalities more than tripled from 2019 to 2021, with fentanyl identified in 77% of all overdose deaths in this age group [31]. Notably, most overdose deaths among adolescents are unintentional [32]. Our study found a surprisingly low number of survey respondents provide naloxone for an opioid-related visit. Naloxone can play a critical role in curbing opioid overdose deaths, and therefore, further exploration for the lack of naloxone prescriptions among PEM physicians is needed. In addition to naloxone, research suggests that ED-initiated buprenorphine in the adult population leads to both increased engagement in formal substance use disorder treatment and reduced self-reported substance use [33]. Furthermore, interventions such as the addition of electronic referrals to addiction clinics and alerts identifying OUD patient have been implemented in adult EDs and could be adapted and replicated in pediatric settings [34].

Interestingly, despite this resounding evidence in support of ED-initiated buprenorphine, our study found very low completion of the buprenorphine waiver course or interest in becoming waivered. At the time our survey was conducted, physicians had to complete an eight-hour training course in order to apply for a waiver to prescribe buprenorphine. Since that time, a new regulation allows for prescribers to apply for a waiver to treat up to 30 patients at a time [35]. Completion of the course permits providers to treat greater than 30 patients [35]. Furthermore, the American College of Emergency Physician recently released “Consensus Recommendations on the Treatment of Opioid Use Disorder” that states that buprenorphine should be considered for adolescents 16 years and older with opioid use disorder [36]. While these guidelines are important resources for pediatric physicians, further clinical guidelines or practice recommendations are needed for PEM physicians. This public health emergency demands further transformative change and rational policies in the treatment of OUD.

Nonfatal opioid overdose is a significant risk factor for subsequent fatal overdose: those who survive an opioid overdose are 100 times more likely to die by drug overdose in the following year [36]. Initiation of buprenorphine in the ED has been associated with decreased drug use and decreased mortality [36], and is therefore a potent intervention for PEM physicians to provide. We recognize that in addition to further education, curricula, and protocols on substance use in the pediatric ED, future efforts are needed to help motivate and empower PEM physicians that this is within their scope of practice.

With increasing presentations of adolescent substance use occurring in pediatric emergency departments, PEM physicians are likely to see more issues related to both acute intoxication and withdrawal, which can lead to a range of morbidity and mortality. The development of substance use curricula and protocols as well as improved initiation of medication for addiction treatment such as buprenorphine may help PEM physicians more appropriately manage adolescent substance use in the ED setting.

## Limitations

This study has several limitations. The primary limitation of our survey was the low response rate, despite monthly survey reminders to the AAP PEM-CRC Listserv members. While this response rate is similar to that reported in previous studies utilizing this Listserv [37, 38], as well as other web-based survey studies of pediatric emergency physicians [39], the high non-response rate may have introduced self-selection bias to those interested in substance use. Second, recruitment for this survey entailed email to members of AAP PEM-CRC listserv, which may be a biased sample. Most respondents were academic, university-based physicians in urban settings, which may limit the generalizability of our results. This is particularly relevant given that most pediatric emergency care in the US is provided by emergency physicians without specialized pediatric training [40]. Furthermore, the average age of respondents is likely higher than the overall age of practicing PEM physicians. Older physicians may have differing levels of comfort in treating substance use given the changes in training over the years. Third, we did not restrict the number of respondents per site, which may have biased our responses to reflect patterns at institutions with larger number of respondents. Finally, as with all surveys, response bias may have occurred. It is possible that respondents may have answered questions the way they thought the surveyors wanted them to rather than based off of their true knowledge and practice.

## Conclusions

Emergency department visits can be a critical touchpoint for adolescent patients with substance use related presentations. For some youth, the PED may be the only point of contact with the health care system. PEM physicians have an opportunity to play a vital role in the ongoing care of adolescents with substance use disorder to minimize the potential harms associated with substance use and support treatment initiation. This study suggests that many PEM physicians surveyed lack comfort in treating certain aspects of substance use, despite increasing presentations for both opioid and non-opioid substance use in pediatric emergency departments. Physicians recognized their limited comfort in managing substance use and expressed interest in additional education and training to improve patient care. Future interventions should focus on the development of curricula and clinical guidelines for managing substance use in PEDs and initiation and referral to treatment in the PED. Specific efforts aimed at increasing the use of medication for addiction treatment such as buprenorphine for opioid use disorder among PEM physicians are critical in addressing this public health crisis.

## Supplementary Information

**Additional file 1.** Pediatric Emergency Medicine Collaborative Research Committee (PEM-CRC) Survey.

**Additional file 2.** Characteristic of the cohort of physicians interested in becoming buprenorphine waivered.

## Data Availability

The datasets used and/or analyzed during the current study are available from the corresponding author on reasonable request.

## Abbreviations

PED: Pediatric Emergency Department
ED: Emergency Department
PEM: Pediatric Emergency Medicine
AAP: American Academy of Pediatrics
PEM-CRC: Pediatric Emergency Medicine Collaborative Research Committee
SOEM: Section on Emergency Medicine
US: United States
SUD: Substance use disorder
OUD: Opioid use disorder
