# Supplementary material for: Attitudes and training related to substance use in pediatric emergency departments
Source: Addict Sci Clin Pract. 2022 Oct 23;17:59. doi: 10.1186/s13722-022-00339-w (PMC9590142; doi:10.1186/s13722-022-00339-w)
Supplement: Supplementary file 1 — Additional file 1. Pediatric Emergency Medicine Collaborative Research Committee (PEM-CRC) Survey. [file 13722_2022_339_MOESM1_ESM.pdf]

PEMCRC Survey

Please complete the survey below.

Thank you!

You are being asked to voluntarily participate in a research study. We are doing this study to assess what protocols and education related to adolescent substance use currently exist in pediatric emergency departments (PED) across the United States. If you agree, we will ask you to complete the 10-minute survey that follows.

Although you will have the option to provide your name and contact information so that you can be entered in a drawing to win one of four $300 cash card prizes, your answers will remain confidential and all study results will only be reported in aggregate. You will not be signing this form.

If you have any questions about this study, please contact Dr. Sarah Bagley at sarah.bagley@bmc.org or 617-414-6906.

Introduction
This brief survey will ask questions about adolescent substance use. In this survey, please consider 'adolescents' to be patients 12-21 years old.

Some questions refer to use of 'non-opioid substances'. For these questions, please consider use of tobacco, alcohol, marijuana, cocaine, or other non-opioid substances. Do not consider opioids in answering these questions.

Other questions refer to 'non-medical opioid use'. For these questions, please consider heroin, illicit fentanyl, or prescription opioids, such as oxycodone, hydrocodone, hydromorphone, methadone, codeine, and others.

Section A: Practice Setting

A1. What is your primary hospital site?

- ◯ University-affiliated
- ◯ Community-based
- ◯ Military
- ◯ Other

Other primary hospital site: ______________________________

A2. What is your primary practice site?

- ◯ Pediatric emergency department in a children's hospital
- ◯ Pediatric emergency department in a general hospital
- ◯ General emergency department
- ◯ Pediatric urgent care
- ◯ Other

Other primary practice site: ______________________________

A3. What setting is your primary practice site in?

- ◯ Urban
- ◯ Suburban
- ◯ Rural

A4. What is your annual ED pediatric patient volume?

- ◯ < 20,000
- ◯ 20,000 - 39,999
- ◯ 40,000 - 59,999
- ◯ >=60,000
- ◯ Don't know

A5. What is your clinical role in your primary practice site?

- ◯ Attending physician
- ◯ Fellow
- ◯ Other

Other clinical role: __________________________

Section B: Procedures/Protocols

B1. In your state, are physicians mandated to complete safer opioid prescribing training?

- ◯ Yes
- ◯ No
- ◯ Unsure

For the following questions, please consider the setting where you primarily practice, the place where you spend the majority of your clinical time.

B2. Please provide your best estimate of how often you take care of adolescent patients who present with a chief complaint related to NON-OPIOID SUBSTANCES (i.e. tobacco, alcohol, marijuana, cocaine, or other non-opioid substances).

Examples of chief complaints may be: intoxication, drug poisoning, or alcohol withdrawal.

- ◯ Every day
- ◯ At least once a week but not daily
- ◯ At least once a month but not weekly
- ◯ Less than monthly

B3. Please provide your best estimate of how often you take care of adolescent patients who present with a chief complaint related to NON-MEDICAL OPIOID USE (opioids can include heroin, illicit fentanyl, or prescription opioids)

Examples of chief complaints may be: opioid overdose, opioid withdrawal, requesting treatment for opioid use disorder, and complications of injection drug use.

- ◯ Every day
- ◯ At least once a week but not daily
- ◯ At least once a month but not weekly
- ◯ Less than monthly

B4. In your primary practice site, do you have any protocols for managing adolescents who present with a chief complaint related to ANY substance use (including opioids)? Protocols refer to treatment protocols, clinical practice guidelines, clinical pathways, or other systematic approach to standardize medical management of adolescents with substance use.

Examples include: acute pain management in patients with opioid addiction, and management of acute opioid withdrawal.

- ◯ Yes
- ◯ No
- ◯ Unsure

B5. What specific protocols for medical management do you have? (select ALL that apply). Protocol for:

- ☐ Alcohol withdrawal
- ☐ Opioid withdrawal
- ☐ Nicotine withdrawal
- ☐ Cannabis withdrawal
- ☐ Acute pain management in patients with opioid use disorder
- ☐ Buprenorphine initiation for opioid use disorder

B6. How often do you query your state prescription monitoring database?	◯ Never<br>◯ 1-10 times a month<br>◯ More than 10 times a month<br>◯ My state does not have one
B7. Are patients prescribed naloxone when there is an opioid-related visit? (select ALL that apply)	☐ Yes, naloxone kit given at discharge<br>☐ Yes, prescription written and given to patient/faxed to pharmacy<br>☐ Sometimes<br>☐ No<br>☐ Unsure
B8. Are any of the following consultants available to your primary practice site in person or over the phone to assist in the care of adolescent patients with substance use disorders? (select ALL that apply)	☐ Addiction Medicine Team<br>☐ Pain Team<br>☐ Adolescent Medicine Team<br>☐ Psychiatry Team<br>☐ Addiction Psychiatry Team<br>☐ Medical Toxicology Team<br>☐ Hospital Medicine Team<br>☐ Social Work<br>☐ Other
Other consultant available:	__________________________________
B9. Do you contact Poison Control when managing adolescent patients with complications related to substance use?	◯ Yes<br>◯ No
In order to prescribe buprenorphine per DEA regulations, you have to complete an approved 8-hour training course. This standardized training does not vary between states.<br><br>B10. To your knowledge, is this course offered on-site to physicians?	◯ Yes<br>◯ No<br>◯ Unsure
If yes, have you completed the training course to become a waivered buprenorphine prescriber?	◯ Yes<br>◯ No
Where did you complete training?	◯ Online: 8 hour training<br>◯ Hybrid training: 4 hours online, 4 hours in-person<br>◯ In-person: 8 hour training
Have you obtained your waiver?	◯ Yes<br>◯ No
If no, why not?	☐ Did not finish the paperwork<br>☐ Relevancy of training<br>☐ Forgot<br>☐ Other
Other reason for not obtaining waiver:	__________________________________
Are you interested in training to become a waivered buprenorphine prescriber?	◯ Yes<br>◯ No

B11. What are the barriers to completing training to become a waivered buprenorphine prescriber? (select ALL that apply)

- ☐ Time
- ☐ Availability of training course
- ☐ Cost
- ☐ Relevancy to practice
- ☐ Nursing support
- ☐ Comfort treating opioid use disorder
- ☐ Unfamiliar/unsure of its indications for use
- ☐ Other

Other barrier: __________________________________

B12. Is there an outpatient adolescent substance use treatment program affiliated with your institution?

- ◯ Yes
- ◯ No
- ◯ Unsure

B13. Are there detox centers in your state that accept patients under the age of 18?

- ◯ Yes
- ◯ No
- ◯ Unsure

Section C: Attitudes

C1. To your knowledge, does your institution or emergency department have ANY educational curricula that includes ANY teaching on adolescent SUBSTANCE USE (i.e. grand rounds, case conferences)?

- ◯ Yes
- ◯ No
- ◯ Unsure

C2. How important is it for pediatric ED physicians to have education about adolescent use of NON-OPIOID SUBSTANCES (i.e. tobacco, alcohol, marijuana, cocaine, or other non-opioid substances)?

- ◯ Very important
- ◯ Important
- ◯ Somewhat important
- ◯ Not important

C3. How important is it for pediatric ED physicians to have education about adolescent NON-MEDICAL OPIOID USE (opioids can include heroin, illicit fentanyl, or prescription opioids)?

- ◯ Very important
- ◯ Important
- ◯ Somewhat important
- ◯ Not important

C4. How would you rate your comfort with the following when providing clinical care for adolescents who present with substance use?

	Very comfortable	Comfortable	Neither comfortable or uncomfortable	Not comfortable
Major medical complications of substance use (i.e. endocarditis, cellulitis)	○	○	○	○
Additional medical screening that may be indicated if chief complaint is related to substance use (i.e. HIV testing or Hepatitis C screening)	○	○	○	○
Acute treatment of intoxication	○	○	○	○
Acute treatment of withdrawal	○	○	○	○
Treatment referral for substance use disorder	○	○	○	○

C5. How important is it for pediatric ED physicians to receive training for the following complications with adolescent patients?

	Very important	Important	Neither important or unimportant	Not important
Major medical complications of substance use (i.e. endocarditis, cellulitis)	○	○	○	○
Additional medical screening that may be indicated if chief complaint is related to substance use (i.e. HIV testing or Hepatitis C screening)	○	○	○	○
Acute treatment of intoxication	○	○	○	○
Acute treatment of withdrawal	○	○	○	○
Treatment referral for substance use disorder	○	○	○	○

C6. In your primary practice site, what are the barriers to receiving physician education on adolescent opioid misuse or opioid addiction (heroin, illicit fentanyl, or prescription opioids)? (select ALL that apply)

- ☐ Lack of faculty expertise
- ☐ Lack of faculty interest
- ☐ Lack of resources
- ☐ Lack of available curricular content
- ☐ Lack of curricular time
- ☐ Lack of adolescent patients who use substances
- ☐ Perceived difficulty of working with adolescents who misuse opioids
- ☐ Unsure
- ☐ Other

Other barrier to providing physician education: ______________________

C7. Would you be interested in a free, shared, web-based curriculum on adolescent opioid misuse (heroin, illicit fentanyl, or prescription opioids) and related topics?

◯ Yes
◯ No
◯ Maybe

C8. Is your primary practice site a training site for residents and/or fellows?

◯ Yes
◯ No

C9. Does the PED curriculum for trainees at your institution include any teaching on adolescent substance use (i.e. tobacco, alcohol, marijuana, cocaine, heroin, illicit fentanyl, prescription opioids, or other substances)?

◯ Yes, for residents only
◯ Yes, for fellows only
◯ Yes, for both residents and fellows
◯ No curriculum on adolescent substance use for residents or fellows
◯ Unsure

C10. We appreciate your perspective. Thank you for your participation in our survey.

Please use the below space if you would like to share any other thoughts about your experience learning about substance use or managing youth with substance use disorders.
We also welcome your feedback on this survey.

____

Section D: Demographics

D1. Would you describe yourself as:

◯ Male
◯ Female
◯ Male to Female Transgender Person
◯ Female to Male Transgender Person
◯ Genderqueer
◯ Other (please specify)

Other gender:

____

D2. What sex were you assigned at birth, on your original birth certificate?

◯ Male
◯ Female

D3. What is your age?

◯ < 30 years old
◯ 30-40 years old
◯ 40-50 years old
◯ > 50 years old

D4. How many years have you been in independent clinical practice (excluding fellowship)?

◯ < 5
◯ 5-9
◯ 10-15
◯ > 15

D5. What is your board certification? (select ALL that apply)

☐ Pediatrics
☐ Emergency medicine
☐ Pediatric emergency medicine
☐ Other
☐ None

Other board certification:

____

D6. What state is your PED located in?

- ◯ AL Alabama
- ◯ AK Alaska
- ◯ AS American Somoa
- ◯ AZ Arizona
- ◯ AR Arkansas
- ◯ CA California
- ◯ CO Colorado
- ◯ CT Connecticut
- ◯ DE Delaware
- ◯ DC District of Columbia
- ◯ FM Federated States of Micronesia
- ◯ FL Florida
- ◯ GA Georgia
- ◯ GU Guam
- ◯ HI Hawaii
- ◯ ID Idaho
- ◯ IL Illinois
- ◯ IN Indiana
- ◯ IA Iowa
- ◯ KS Kansas
- ◯ KY Kentucky
- ◯ LA Louisiana
- ◯ ME Maine
- ◯ MH Marshall Islands
- ◯ MD Maryland
- ◯ MA Massachusetts
- ◯ MI Michigan
- ◯ MN Minnesota
- ◯ MS Mississippi
- ◯ MO Missouri
- ◯ MT Montana
- ◯ NE Nebraska
- ◯ NV Nevada
- ◯ NH New Hampshire
- ◯ NJ New Jersey
- ◯ NM New Mexico
- ◯ NY New York
- ◯ NC North Carolina
- ◯ ND North Dakota
- ◯ MP Northern Mariana Islands
- ◯ OH Ohio
- ◯ OK Oklahoma
- ◯ OR Oregon
- ◯ PW Palau
- ◯ PA Pennsylvania
- ◯ PR Puerto Rico
- ◯ RI Rhode Island
- ◯ SC South Carolina
- ◯ SD South Dakota
- ◯ TN Tennessee
- ◯ TX Texas
- ◯ UT Utah
- ◯ VT Vermont
- ◯ VI Virgin Islands
- ◯ VA Virginia
- ◯ WA Washington
- ◯ WV West Virginia
- ◯ WI Wisconsin
- ◯ WY Wyoming
- ◯ Other

Other state: __________________________________

D7. In what county (e.g., Miami-Dade county) is your clinical setting located? (Please do not provide your country) __________________________________

Thank you for your participation. When you click "submit" you will be automatically directed to a separate page where you can input your name and contact information for entry into the survey raffle.

We've done this to save your identifying information separately from your survey responses.
