# Supplementary material for: Attitudes and training related to substance use in pediatric emergency departments
Source: Addict Sci Clin Pract. 2022 Oct 23;17:59. doi: 10.1186/s13722-022-00339-w (PMC9590142; doi:10.1186/s13722-022-00339-w)
Supplement: Supplementary file 2 — Additional file 2. Characteristic of the cohort of physicians interested in becoming buprenorphine waivered. [file 13722_2022_339_MOESM2_ESM.docx]

**Table 3.** Characteristics of the cohort of physicians interested in becoming buprenorphine waivered

|  | Total  n (%) | Interested  n (%) | Not interested  n (%) | P-value^*^ |
| --- | --- | --- | --- | --- |
| Overall number | 163 | 44 (27.0) | 119 (73.0) |  |
| Gender, n (%) | | | | |
| Male | 72 (44.2) | 17 (38.6) | 55 (46.2) | 0.39 |
| Female | 91 (55.8) | 27 (61.4) | 64 (53.8) |  |
| Age, n (%) | | | | |
| 30 – 40 years | 37 (22.7) | 16 (36.4) | 21 (17.7) | 0.04 |
| 41 – 50 years | 53 (32.5) | 12 (27.3) | 41 (34.5) |  |
| > 50 years | 73 (44.8) | 16 (36.4) | 57 (47.9) |  |
| Region, n (%) | | | | |
| Northeast | 37 (22.7) | 10 (22.7) | 27 (22.7) | 0.29 |
| South | 41 (25.2) | 8 (18.2) | 33 (27.7) |  |
| Midwest | 48 (29.5) | 12 (27.3) | 36 (30.3) |  |
| West | 35 (21.5) | 14 (31.8) | 21 (17.7) |  |
| Other | 2 (1.2) | 0 (0.0) | 2 (1.7) |  |
| Hospital site, n (%) | | | | |
| University affiliated | 142 (87.1) | 37 (84.1) | 105 (88.2) | 0.48 |
| Community based/other | 21 (12.9) | 7 (15.9) | 14 (11.8) |  |
| Annual ED volume, n (%) | | | | |
| 0 – <40,000 | 49 (30.1) | 14 (31.8) | 35 (29.4) | 0.77 |
| ≥ 40,000 | 114 (69.9) | 30 (68.2) | 84 (70.6) |  |

^*^ P values are a comparison between the Interested and Not interested responses calculated employing the Pearson’s chi-square test. The P value of significance for this table is set at 0.05.
